# β-aminoisobutyric acid attenuates hepatic endoplasmic reticulum stress and glucose/lipid metabolic disturbance in mice with type 2 diabetes

**DOI:** 10.1038/srep21924

**Published:** 2016-02-24

**Authors:** Chang-Xiang Shi, Ming-Xia Zhao, Xiao-Dong Shu, Xiao-Qing Xiong, Jue-Jin Wang, Xing-Ya Gao, Qi Chen, Yue-Hua Li, Yu-Ming Kang, Guo-Qing Zhu

**Affiliations:** 1 Key Laboratory of Cardiovascular Disease and Molecular Intervention, Department of Physiology, Nanjing Medical University, Nanjing, Jiangsu 210029, China; 2 Department of Pathophysiology, Nanjing Medical University, Nanjing, Jiangsu 210029, China; 3 Department of Physiology and Pathophysiology, Cardiovascular Research Center, Xi’an Jiaotong University School of Medicine, Xi’an 710061, China

## Abstract

β-aminoisobutyric acid (BAIBA) is a nature thymine catabolite, and contributes to exercise-induced protection from metabolic diseases. Here we show the therapeutical effects of BAIBA on hepatic endoplasmic reticulum (ER) stress and glucose/lipid metabolic disturbance in diabetes. Type 2 diabetes was induced by combined streptozotocin (STZ) and high-fat diet (HFD) in mice. Oral administration of BAIBA for 4 weeks reduced blood glucose and lipids levels, hepatic key enzymes of gluconeogenesis and lipogenesis expressions, attenuated hepatic insulin resistance and lipid accumulation, and improved insulin signaling in type 2 diabetic mice. BAIBA reduced hepatic ER stress and apoptosis in type 2 diabetic mice. Furthermore, BAIBA alleviated ER stress in human hepatocellular carcinoma (HepG2) cells with glucosamine-induced insulin resistance. Hepatic AMPK phosphorylation was reduced in STZ/HFD mice and glucosamine-treated HepG2 cells, which were restored by BAIBA treatment. The suppressive effects of BAIBA on glucosamine-induced ER stress were reversed by knockdown of AMPK with siRNA. In addition, BAIBA prevented thapsigargin- or tunicamycin-induced ER stress, and tunicamycin–induced apoptosis in HepG2 cells. These results indicate that BAIBA attenuates hepatic ER stress, apoptosis and glucose/lipid metabolic disturbance in mice with type 2 diabetes. AMPK signaling is involved to the role of BAIBA in attenuating ER stress.

Endoplasmic reticulum (ER) is a central organelle for protein synthesis and folding^1^. Under physiologic conditions, there is an equilibrium between the protein synthesis and its folding capacity, and these proteins are correctly folded and assembled by chaperones in the ER. When the protein synthesis rate exceeds the folding capacity, unfolded proteins accumulate in ER^2^. The perturbation in ER homeostasis causes ER stress and leads to activation of the complex signaling cascade called the unfolded protein response (UPR)^1^. Activation of UPR signaling causes a transcriptional activation of ER chaperones and a reduction in protein synthesis, which aid in restoring the ER homeostasis^3^. When the UPR fails to restore the ER homeostasis, the cells may undergo a pro-apoptotic ER stress response pathway^4^.

ER stress is a crucial link among obesity, insulin resistance and diabetes^5^. It is closely associated with hepatic insulin resistance and plays important roles in altered glucose homoeostasis in type 2 diabetes^6^. Obesity causes ER stress, hepatic steatosis and insulin resistance^7^. Obesity-induced ER stress inhibits insulin signaling via hyperactivation of c-Jun N-terminal kinase (JNK) and subsequent serine phosphorylation of insulin receptor substrate-1 (IRS-1)^8^. ER stress has been proposed as a novel mechanism for the development of insulin resistance in obesity^9^. Insulin signaling in liver is attenuated in diabetes-evoked ER stress, indicated by reduced Akt phosphorylation and IRS-1 tyrosine phosphorylation^10^. Inhibition of cytochrome P450 4A reduces hepatic ER stress and insulin resistance in mice^9^. Similarly, chemical chaperones reduce ER stress and restore glucose homeostasis in type 2 diabetes^5^. ER stress plays a crucial role in the insulin resistance and could be a potential therapeutic target for diabetes^10^.

β-aminoisobutyric acid (BAIBA) is a nonprotein β-amino acid, a catabolite of thymine, which is further degraded into propionyl-CoA, methylmalonyl-CoA and succinyl-CoA within mitochondria, especially in liver^11^,^12^. BAIBA can also be generated by catabolism of the branched-chain amino acid valine^13^. BAIBA reduces body fat percentage through increased fatty acid oxidation (FAO) and decreased de novo lipogenesis in liver in mice^14^. Recently, BAIBA is identified as a small molecule myokine secreted from myocytes with forced expression of peroxisome proliferator-activated receptor gamma coactivator-1α (PGC-1α)^13^. BAIBA enhances the browning of white adipose tissue and FAO in the liver via peroxisome proliferator-activated receptor α (PPARα), and may contribute to exercise-induced protection from metabolic diseases^13^. More recently, it has been found that BAIBA attenuates insulin resistance, inhibits inflammation and induces FAO in skeletal muscle via AMPK-PPARδ pathway^15^. However, whether BAIBA is involved in ER stress remains unknown. In the present study, the therapeutic effects of BAIBA on hepatic ER stress, apoptosis and glucose/lipid metabolic disturbance in type 2 diabetes were investigated.

## Results

### General data

**Figure 1:**
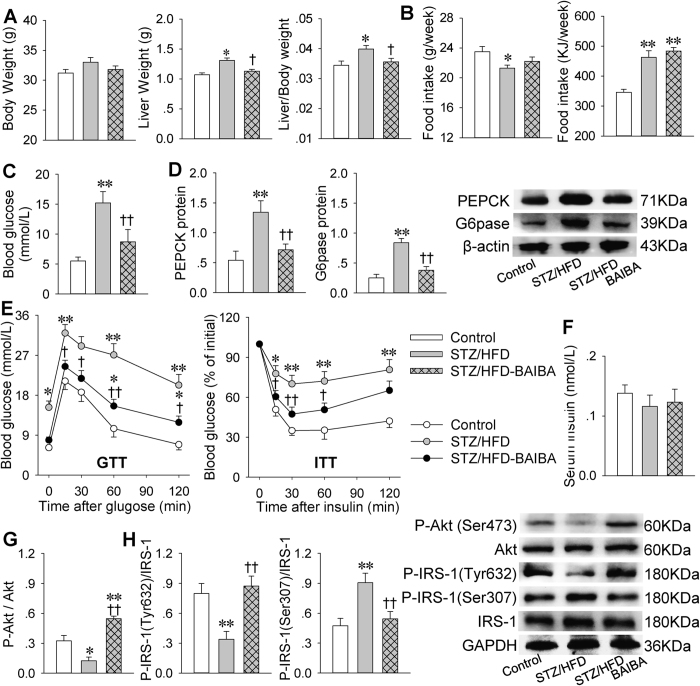
BAIBA attenuates glucose metabolic disturbance and improves insulin resistance in STZ/HFD-induced type 2 diabetes in mice. (**A**), body weight, liver weight and the ratio of liver weight to body weight. (**B**), Accumulated food intake in the last week (g/week and KJ/week). (**C**), fasting blood glucose levels. (**D**), expressions of key enzymes of gluconeogenesis (PEPCK, G6pase) in livers. (**E**), glucose tolerance test (GTT) and insulin tolerance test (ITT). (**F**), serum insulin levels. (**G**), phosphorylation of Akt. (**H**), phosphorylation of insulin receptor substrate-1 (IRS-1) at Tyr632 and at Ser307. *P < 0.05 and **P < 0.01 vs. Control, ^†^P < 0.05 and ^††^P < 0.01 vs. STZ/HFD. n = 7 for each group in (**A–C,E,F**); n = 4 for each group in (**D, G, H**).

Liver weight and the ratio of liver weight to body weight were increased in streptozotocin/high-fat diet (STZ/HFD) mice compared with control mice, which were prevented by BAIBA. However, there were no significant difference in body weight and food intake between STZ/HFD mice and STZ/HFD-BAIBA mice (Fig. 1A,B).

### BAIBA attenuates glucose metabolic disturbance and insulin resistance in diabetic mice

Fasting blood glucose levels were increased in STZ/HFD mice, which were reduced by BAIBA (Fig. 1C). Hepatic glucose 6-phosphatase (G6pase) and phosphorenol pyruvate carboxy-kinase (PEPCK), two key enzymes of gluconeogenesis^16^, were upregulated in STZ/HFD mice, which were attenuated by BAIBA (Fig. 1D). Insulin tolerance test (ITT) and glucose tolerance test (GTT) were used for evaluating insulin resistance and glucose tolerance. BAIBA not only restored the impaired glucose tolerance but also improved insulin resistance in STZ/HFD mice (Fig. 1E). To determine whether insulin would be involved in the effects of BAIBA, serum insulin levels and insulin signaling were examined. There were no significant difference among Control, STZ/HFD and STZ/HFD-BAIBA mice (Fig. 1F). The phosphorylation of Akt (Ser473) and IRS-1 (Tyr632) were reduced but the phosphorylation of IRS-1 (Ser307) was increased in STZ/HFD mice, which were restored by BAIBA (Fig. 1G,H). These results indicate that the BAIBA reduces blood glucose and hepatic gluconeogenesis and improves hepatic insulin resistance in type 2 diabetes, which is associated with the improvement of insulin signaling rather than the circulating insulin levels.

### BAIBA alleviates lipid metabolic derangements in diabetic mice

**Figure 2:**
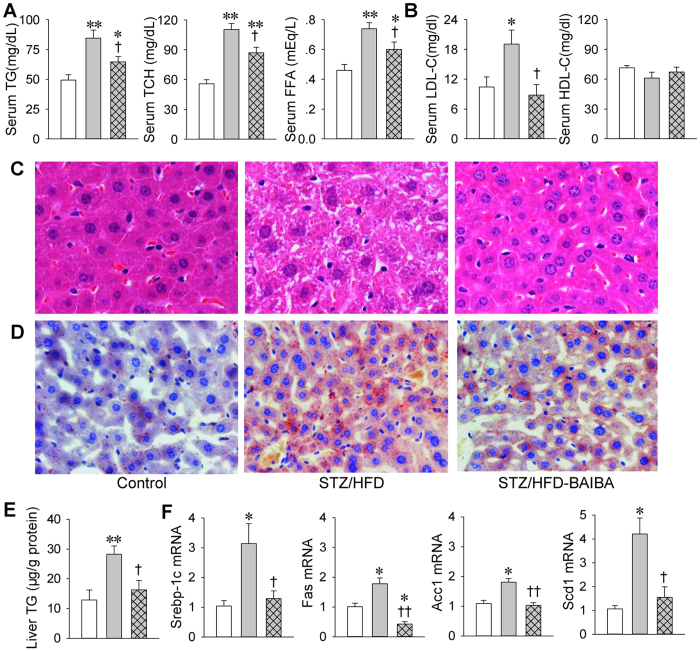
BAIBA attenuates lipid metabolic derangements in mice with type 2 diabetes. (**A**), Serum triglyceride (TG), total cholesterol (TCH) and free fatty acid (FFA) levels. (**B**), Serum low density lipoprotein cholesterol (LDL-C) and high density lipoprotein cholesterol (HDL-C) levels. (**C**), HE staining of liver sections. (**D**), Oil red O staining of liver sections. (**E**), Liver triglyceride (TG) levels. (**F**), Expressions of lipogenesis genes (Srebp-1c, Fas, Acc1, Scd1) in livers. *P < 0.05 and **P < 0.01 vs. Control; ^†^P < 0.05 and ^††^P < 0.01 vs. STZ/HFD. n = 7 for each group in (**A,B,E**); n = 4 for each group in (**C,D,F**).

Serum triglyceride (TG), total cholesterol (TCH) and free fatty acid (FFA) levels were increased in STZ/HFD mice, which were attenuated by BAIBA (Fig. 2A). Moreover, serum low-density lipoprotein cholesterol (LDL-C) levels were increased in STZ/HFD mice, which were prevented by BAIBA (Fig. 2B). In consonance with the hypolipidemic effect of BAIBA, STZ/HFD-induced hepatic steatosis were attenuated by BAIBA, as evidenced by the liver sections with HE staining (Fig. 2C) and oil red O staining (Fig. 2D) as well as liver TG contents (Fig. 2E). As hepatic *de novo* lipogenesis is an important factor contributing to hepatic lipid accumulation^17^, we further observed the effects of BAIBA on the key markers of lipogenesis including sterol regulatory element-binding protein-1c (Srebp-1c), fatty acid synthase (Fas), acetyl-CoA carboxylase 1 (Acc1) and stearoyl-CoA desaturase 1 (Scd1). The up-regulation of hepatic Srebp-1c, Fas, Acc1 and Scd1 mRNA expressions were abolished by BAIBA (Fig. 2F). These results indicate that BAIBA attenuates hyperlipoidemia and hepatic steatosis and reduces hepatic *de novo* lipogenesis in type 2 diabetes.

### BAIBA reduces hepatic ER stress and apoptosis in diabetes mice

**Figure 3:**
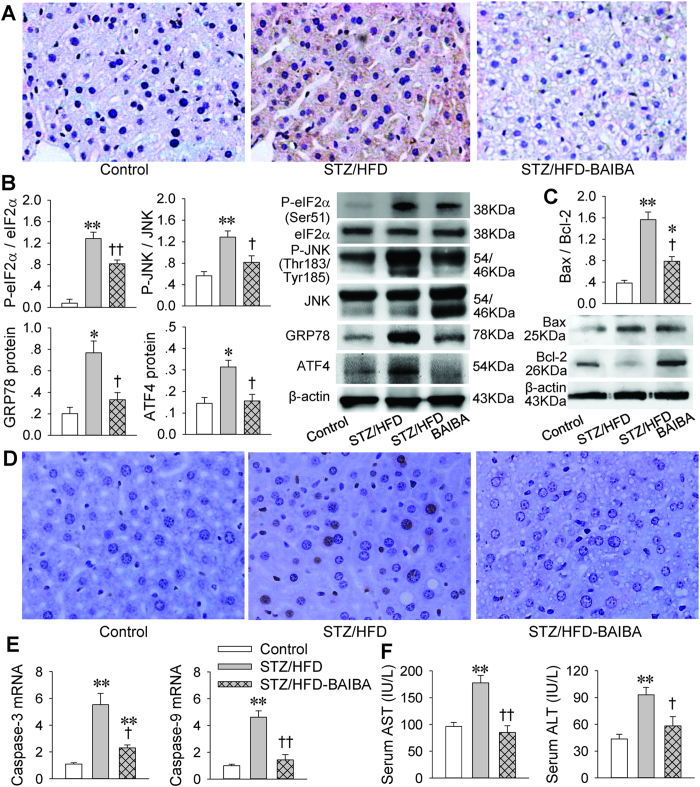
BAIBA improves hepatic ER stress in type 2 diabetes of mice. (**A**), Immunohistochemistry of liver sections for CHOP (an ER stress marker). (**B**), Western blot analysis for phosphorylation of eIF2α and JNK, and expression of GRP78 and ATF4 protein (ER stress markers) in liver. (**C**) Expression of Bax and Bcl-2 proteins and ratio of Bax to Bcl-2 (apoptosis markers) in liver. (**D**), TUNEL staining showing apoptosis in liver. (**E**), Expression of caspase-3 and caspase-9 (apoptosis markers) in liver. (**F**), Serum ALT and AST levels (markers of hepatic injury). *P < 0.05 and **P < 0.01 vs. Control, ^†^P < 0.05 and ^††^P < 0.01 vs. STZ/HFD. n = 4 for each group in (**A–D**); n = 7 for each group in (**E,F**).

ER stress plays a crucial role in the insulin resistance found in diabetes and thus could be a potential therapeutic target for diabetes^8^,^10^. Glucose-regulated protein 78 (GRP78) is a well-known ER stress chaperone^18^. The dissociation of GRP78 from protein kinase R-like ER kinase (PERK) initiates the dimerization and autophosphorylation of the kinase, and generates active PERK, and then causes eukaryotic initiation factor 2 (eIF2) phosphorylation^19^. It has been found that the downstream signaling activity of PERK is enhanced in the livers of diabetic mice, reflected in the phosphorylation of eIF2α, expression of activating transcription factor (ATF) 4 and CCAAT/enhancer-binding protein homologous protein (CHOP)^20^. It is known that CHOP is an important element of the switch from pro-survival to pro-death signaling^19^. Immunohistochemistry showed that CHOP expression in the liver was upregulated in STZ/HFD mice, which was attenuated by BAIBA (Fig. 3A). STZ/HFD induced the upregulation of GRP78 and ATF4 as well as the phosphorylation of eIF2α and JNK in the livers were prevented by BAIBA (Fig. 3B).

Accumulation of unfolded proteins in the ER is toxic to cells. CHOP is a major regulator of ER stress-induced apoptosis^18^. Therefore, the anti-apoptotic and hepatic protective effects of BAIBA were further investigated. Bax is a pro-apoptotic protein, while Bcl-2 is an anti-apoptotic protein which inhibits caspases^21^. The ratio of Bax to Bcl-2 and the mRNA levels of caspase-3 and caspase-9 were increased in STZ/HFD mice, which were reduced by BAIBA (Fig. 3C,E). TUNEL staining showed that the number of apoptotic cells was increased in the liver of STZ/HFD-induced type 2 diabetic mice, which was attenuated by BAIBA treatment (Fig. 3D). Furthermore, serum alanine aminotransferase (ALT) and aspartate aminotransferase (AST) levels, the liver injury markers, were increased in STZ/HFD mice, which were restored by BAIBA (Fig. 3F). These results indicate that BAIBA improves diabetes in STZ/HFD induced diabetic mice by attenuating ER stress and inhibiting apoptosis in the liver. These results indicate BAIBA plays crucial anti-apoptotic and hepatic protective roles in type 2 diabetes.

### BAIBA attenuates glucosamine-induced ER stress in HepG2

**Figure 4:**
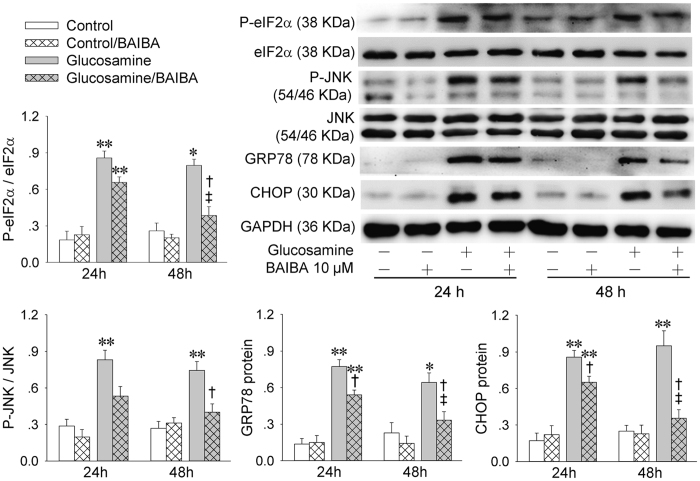
BAIBA improves glucosamine-induced ER stress in HepG2 cells. The HepG2 cells were treated with glucosamine (18 mM) for 18 h, and then treated with BAIBA (10 μM) for 24 h or 48 h. The phosphorylation of eIF2α and JNK, and the expressions of GRP78 and CHOP were used as the markers of ER stress. *P < 0.05 and **P < 0.01 vs. Control; ^†^P < 0.05 vs. Glucosamine. ^‡^P < 0.05 vs. 24 h. n = 4 for each group.

High concentration of glucosamine attenuates metabolic signaling of insulin, and is generally used to induce insulin resistance and ER stress *in vivo*^22^,^23^,^24^. We sought to determine whether BAIBA treatment could reduce ER stress in HepG2 cells with glucosamine-induced insulin resistance. Glucosamine increased the eIF2α and JNK phosphorylation, and upregulated the GRP78 and CHOP protein expressions in HepG2 cells, which were prevented by BAIBA (Fig. 4). These results indicate that BAIBA attenuates glucosamine-induced hepatic ER stress *in vivo*.

### BAIBA attenuates thapsigargin-, tunicamycin- or high glucose-induced ER stress in HepG2

**Figure 5:**
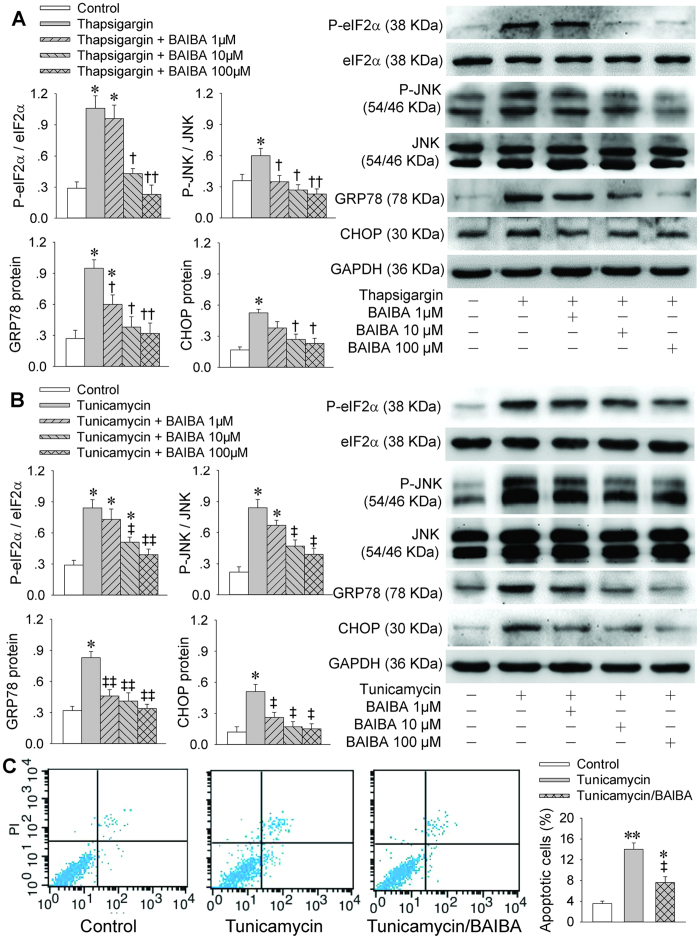
BAIBA improves thapsigargin- or tunicamycin-induced ER stress in HepG2 cells. HepG2 cells were treated with thapsigargin (1 μM) or tunicamycin (3 μM) and different doses of BAIBA (1, 10 or 100 μM) for 48 h, followed with thapsigargin (1 μM) or tunicamycin (3 μM) for 4 h. The phosphorylation of eIF2α and JNK, and the expressions of GRP78 and CHOP were used as the markers of ER stress. (**A**), Effect of BAIBA on thapsigargin-induced ER stress. (**B**), Effect of BAIBA on tunicamycin-induced ER stress. (**C**), Effect of BAIBA on apoptosis in tunicamycin-induced apoptosis determined with flow cytometric analysis in HepG2 cells. HepG2 cells were incubated with tunicamycin (3 μM) for 4 h, then treated with BAIBA (100 μM) for 48 h. *P < 0.05 and **P < 0.01 vs. Control; ^†^P < 0.05 and ^††^P < 0.01 vs. thapsigargin; ^‡^P < 0.05 and ^‡‡^P < 0.01 vs. tunicamycin. n = 4 for each group.

An interesting question is whether the role of BAIBA in attenuating ER stress is not only limited to the insulin resistance-related ER stress, but also applied to some other factors-induced ER stress. Thus, we further investigated the effects of BAIBA on the ER stress in other commonly used cellular models. Thapsigargin is an inhibitor of ubiquitous sarco-endoplasmic reticulum Ca^2+^-ATPases (SERCA), which interfere with Ca^2+^balance and induces apoptosis^25^. Tunicamycin is an inhibitor of protein N-linked glycosylation, which specifically inhibits the dolichol pyrophosphate-mediated glycosylation of asparagine residues in nascent glycoproteins, resulting in UPR activation and ER stress^26^. Either thapsigargin or tunicamycin induces ER stress is involved in PERK/eIF2α^27^. We found that either thapsigargin or tunicamycin increased the eIF2α and JNK phosphorylation, and upregulated the GRP78 and CHOP protein expressions in HepG2 cells, which were dose-dependently attenuated by BAIBA. High concentration of BAIBA almost abolished the effects of thapsigargin or tunicamycin (Fig. 5A,B). Flow cytometric analysis showed that BAIBA reduced apoptotic cells in tunicamycin-induced apoptosis in HepG2 cells (Fig. 5C). Moreover, high glucose-induced ER stress in HepG2 cells *in vitro* was used to mimic the hyperglycemia in type 2 diabetic mice. We found that high glucose promoted the eIF2α phosphorylation and upregulated the GRP78 protein expressions in HepG2 cells, which were prevented by BAIBA (Supplementary Figure 1). These results indicate that BAIBA prevented thapsigargin- tunicamycin- or high glucose-induced ER stress *in vivo*.

### BAIBA alleviates tunicamycin-induced ER stress in mice

Tunicamycin was used to induce the hepatic ER stress in mice^28^. The eIF2α phosphorylation and ATF4 expression were increased in the liver of tunicamycin-treated mice, which was restored by the pretreatment with BAIBA (Supplementary Figure 2). The results indicate that BAIBA prevented tunicamycin-induced ER stress *in vitro*.

### AMPK in the role of BAIBA in attenuating ER stress

**Figure 6:**
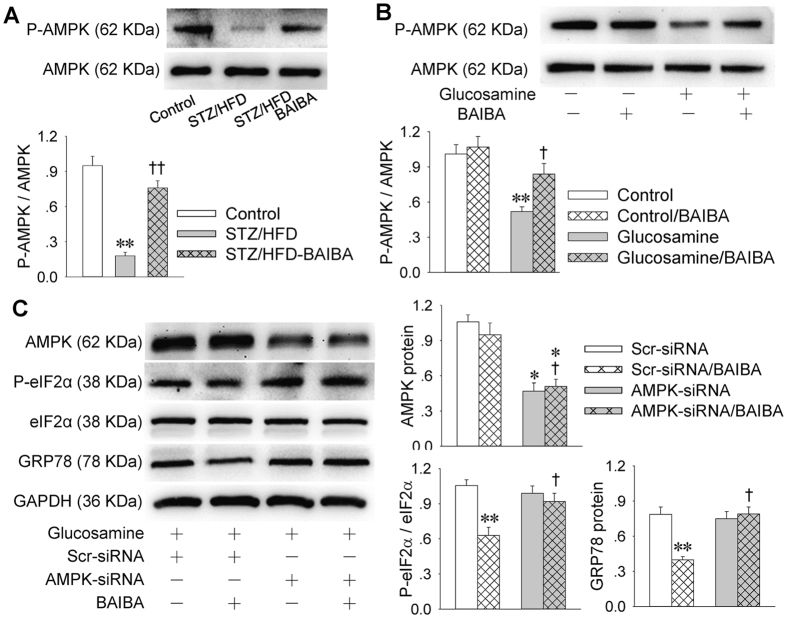
AMPK mediated the role of BAIBA in attenuating ER stress. (**A**), Effect of BAIBA on AMPK phosphorylation in livers of STZ/HFD mice. **P < 0.01 vs. Control; ^††^P < 0.01 vs. STZ/HFD; (**B**), Effect of BAIBA on AMPK phosphorylation in glucosamine-treated HepG2 cells. **P < 0.01 vs. Control; ^†^P < 0.05 vs. glucosamine; (**C**), Effects of knockdown of AMPK on AMPK and GRP78 expressions and eIF2α phosphorylation in glucosamine-treated HepG2 cells. HepG2 cells were transfected with scrambled-siRNA (Scr-siRNA) or AMPK-siRNA for 24 h, and then cultured in the presence of glucosamine for 18 h and treated with BAIBA for 48 h. *P < 0.05 and **P < 0.01 vs. Scr-siRNA; ^†^P < 0.05 vs. Scr-siRNA/BAIBA. n = 4 for each group.

It is known that AMPK is a pharmacological target for inhibiting ER stress^29^,^30^,^31^. We found that AMPK phosphorylation is inhibited in the liver of STZ/HFD mice, which was restored by BAIBA (Fig. 6A). Similarly, AMPK phosphorylation is attenuated in HepG2 cells with glucosamine-induced ER stress (Fig. 6B). The inhibitory effects of BAIBA on eIF2α phosphorylation and GRP78 expression were reversed by knockdown of AMPK with siRNA in the glucosamine-induced ER stress in HepG2 cells (Fig. 6C). The effectiveness of AMPK-siRNA was confirmed by the reduced AMPK expression (Fig. 6C).

### BAIBA alleviates D-galactosamine/lipopolysaccharide-induced apoptosis in the liver of mice

The roles of BAIBA in reducing apoptosis was further evaluated in another animal model. Combined D-galactosamine and lipopolysaccharide (GalN/LPS) were used to induce hepatocyte apoptosis in mice^32^. GalN/LPS upregulated the caspase-3 and caspase-9 protein expressions, which were normalized by pretreatment with BAIBA (Supplementary Figure 3). TUNEL staining showed that BAIBA treatment attenuated the number of apoptotic cells in the GalN/LPS-induced apoptosis in the livers of mice (Supplementary Figure 4).

## Discussion

BAIBA is known to convert white adipose tissue into brown adipose tissue, which plays beneficial roles in improving glucose metabolism and insulin sensitivity^13^. Plasma BAIBA levels are increased with exercise and inversely associated with metabolic risk factors in humans. BAIBA may contribute to exercise-induced protection from metabolic diseases^13^. Recently, it has been found that BAIBA attenuates insulin resistance and inflammation in skeletal muscle^15^. The primary novel findings in the present study are that oral administration of BAIBA attenuates hepatic ER stress, apoptosis and glucose/lipid metabolic disturbance in type 2 diabetes.

Efficient functioning of ER is essential for proper cellular activities and survival. ER is an organelle that synthesizes various proteins which are usually correctly folded and assembled by chaperones^3^. Cells cope with ER stress by enhancing protein folding and degradation and down-regulating overall protein synthesis in the ER^33^. Excessive ER stress or insufficient adaptive response to ER stress may result in cell injury^18^,^34^. ER stress is a central feature of peripheral insulin resistance and type 2 diabetes, and intervention of ER stress may offer new opportunities for treating diabetes^8^,^10^,^35^. In the present study, the expression of GRP78, ATF4 and CHOP were up-regulated, and the phosphorylation of eIF2α and JNK were increased in the livers of mice with STZ/HFD-induced diabetes, which were reduced by long-term administration of BAIBA for 4 weeks. *In vitro*, treatment with BAIBA effectively reduced the ER stress in HepG2 cells with glucosamine-induced insulin resistance or in high glucose-treated HepG2 cells, but not in control HepG2 cells. Furthermore, BAIBA attenuates tunicamycin-induced ER stress in the livers of mice. These results indicate that BAIBA effectively attenuates excessive ER stress in type 2 diabetes. On the other hand, the ratio of Bax to Bcl-2, the caspase-3 and caspase-9 mRNA levels and the number of apoptotic cells were increased in the livers of type 2 diabetic mice, which were prevented by BAIBA. BAIBA attenuates GalN/LPS-induced apoptosis in the livers of mice. Moreover, increased serum ALT and AST levels in type 2 diabetic mice were restored by BAIBA. These results indicate that BAIBA attenuates hepatocellular apoptosis and liver injury in type 2 diabetes. It is known that CHOP is a major regulator of ER stress-induced apoptosis^18^. Activated eIF2α-ATF4-CHOP signaling plays a vital role in the development of type 2 diabetic mice^36^,^37^,^38^. Thus, we believe that the effect of BAIBA on ER stress contributes to its anti-apoptotic and hepatic protective roles in type 2 diabetes. In other two commonly used cellular ER stress models which are independent on insulin resistance^25^,^26^. BAIBA also attenuates ER stress in thapsigargin- or tunicamycin-treated HepG2 cells. These results suggest that the role of BAIBA in attenuating ER stress may be independent on the improvement of insulin resistance, and BAIBA may be used as an effective agent to alleviate ER stress induced by various factors.

It has been found that BAIBA attenuates insulin resistance, suppresses inflammation and induces fatty acid oxidation in skeletal muscle in mice fed with HFD^15^. In the present study, long-term administration of BAIBA reduced fasting blood glucose and improved glucose tolerance and insulin sensitivity in mice with STZ/HFD-induced type 2 diabetes, which supports previous findings^15^. Interestingly, we found that BAIBA had no significant effect on serum insulin levels but normalized the reduced Akt and IRS-1 phosphorylation at Tyr632 and the increased IRS-1 phosphorylation at Ser307 in livers of type 2 diabetic mice. Moreover, BAIBA recovered the upregulated the expression of two key enzymes of gluconeogenesis, PEPCK and G6pase, in livers of type 2 diabetic mice. These results suggest that BAIBA-induced improvement of insulin signaling may contribute to its roles in reducing blood glucose and hepatic gluconeogenesis and improving hepatic insulin resistance in type 2 diabetes. Serum FFA levels correlates with nonalcoholic fatty liver disease (NAFLD) and could be used as an indicator for predicting advanced fibrosis in NAFLD patients^39^. BAIBA normalized the increased serum TG, TCH, FFA and LDL-C levels, hepatic lipid accumulation and lipogenesis gene expressions in type 2 diabetic mice. These results indicate that BAIBA reduces hepatic lipogenesis, and attenuates hyperlipemia and hepatic steatosis in diabetes. It is noted that ER stress is involved in glucose/lipid metabolic disturbance^8^,^34^,^40^. BAIBA increase the expression of brown adipocyte-specific genes in white adipocytes and induces a brown adipose-like phenotype in pluripotent stem cells^41^, suggesting that adipose tissue may be another target of BAIBA. The roles of BAIBA in the interaction of ER stress and glucose/lipid metabolic disturbance need to be elucidated in future studies.

Previous studies have shown that activation of AMPK inhibits oxidized LDL-triggered ER stress, and reduction of AMPK increases ER stress and atherosclerosis^30^,^31^. AMPK is a therapeutic target for treatment of insulin resistance and type 2 diabetes^42^. The present study found that AMPK signaling is involved to the role of BAIBA in attenuating ER stress. It has been found that BAIBA prevents diet-induced obesity in mice with partial leptin deficiency^43^ and BAIBA reversed HFD-induced increases in body weight in mice^44^. In the present study, STZ/HFD-induced type 2 diabetic mice did not showed obvious obesity, and BAIBA had no significant effect on body weight in these mice. This discrepancy may attribute to the different animal models. It is possible that BAIBA only reduced the body weight in obesity mice, but not in non-obese 2 diabetic mice. On the other hand, it has been found that BAIBA attenuates hepatic necroinflammation in the diet-induced obesity in mice with partial leptin deficiency^43^, and skeletal muscle inflammation in HFD-induced mice^44^. As BAIBA reduces hepatic ER stress and apoptosis in type 2 diabetic mice and inflammation is closely relevant to ER stress and apoptosis^45^,^46^, we speculate that BAIBA may attenuate hepatic inflammation in type 2 diabetic mice, which need further investigation.

In summary, oral administration of BAIBA attenuates hepatic ER stress, apoptosis and glucose/lipid metabolic disturbance in the mice with STZ/HFD-induced type 2 diabetic mice. BAIBA alleviates glucosamine-, high glucose-, thapsigargin- or tunicamycin-induced ER stress in HepG2 cells. AMPK signaling is involved to the role of BAIBA in attenuating ER stress. Our findings regarding BAIBA provide new insights toward the development of therapeutic agents aimed at effectively reducing hepatic ER stress and glucose/lipid metabolic disturbance in type 2 diabetes or other metabolic disorders.

## Methods

### Mouse model of type 2 diabetes and BAIBA treatment

Eight-week-old male C57BL/6J mice (Comparative Medicine Centre, Yangzhou University, Yangzhou, china) were used for inducing type 2 diabetes. Experiments were approved by the Experimental Animal Care and Use Committee of Nanjing Medical University. The experimental methods were carried out in accordance with the Guide for the Care and Use of Laboratory Animals (NIH publication, 8th edition, 2011). The mice were housed in an environment with controlled temperature and humidity, a 12-h light/dark cycle, and free access to water and diet.

Mouse model of type 2 diabetes was induced in mice by combination of low-dose of STZ and HFD as we reported previously^47^. This animal model simulates natural disease progression and metabolic characteristics typical of type 2 diabetes, and is identified as an ideal animal model of type 2 diabetes^48^,^49^,^50^. Mice were randomly divided into three groups (n = 7 for each group). The mice in one group were used as control; the mice in other two groups were used to induce type 2 diabetes, which received 4-hour’s fasting and subsequent injection of low-dose of STZ (120 mg/kg in 10 mM citrate buffer, pH 4.0, i.p.), and 3 weeks later, normal diet (14.7 kJ/g, 13% of energy as fat) was replaced with HFD (21.8 kJ/g, 60% of energy as fat; D12492, Research Diets, New Brunswick, NJ, USA). Eight weeks after injection of STZ, the mice in two groups were mixed into one group, and then randomly were re-divided into two groups, which respectively received normal drinking water (STZ/HFD group) and 150 mg/kg/day of BAIBA dissolved in drinking water (STZ/HFD-BAIBA group) for 4 weeks^15^. The mice in control received an injection of vehicle (i.p.) and were fed with normal diet (14.7 kJ/g, 13% of energy as fat) throughout the experiment (Supplementary Figure 5). At the end of the experiment, the mice were euthanized with an overdose of pentobarbital sodium (150 mg/kg, i.v.).

### Other mouse models and BAIBA treatment

Two other mouse models were used to examine the effects of BAIBA on ER stress and apoptosis *in vivo*. In tunicamycin-induced ER stress mouse model^28^, mice were injected with saline or BAIBA (300 mg/Kg, i.p.) 0.5 h before DMSO or tunicamycin (2.5 mg/Kg, i.p.). The livers were collected for measurement 2 h after administration of DMSO or tunicamycin. In GalN/LPS-induced apoptosis mouse model^32^, mice were injected with saline or BAIBA (300 mg/Kg, i.p.) 0.5 h before PBS or GalN (700 mg/kg, i.p.) plus LPS (100 μg/kg, i.p.). The livers were collected for measurement 6 h after administration of PBS or GalN/LPS.

### Cell models and treatment

HepG2 cells were incubated in Dulbecco’s modified Eagle’s medium (DMEM) containing 10% fetal bovine serum (FBS) at 37 °C and 5% CO_2_-95% air, and allowed to adhere for 4 hours. To induce insulin resistance, HepG2 cells were incubated with glucosamine (18 mM) for 18 h in serum-free medium, and followed by treatment with BAIBA (10 μM) for 24 or 48 h in the presence of glucosamine^22^,^23^. To mimic the hyperglycemia in type 2 diabetic mice, high glucose was used to induce ER stress in HepG2 cells^24^. The HepG2 cells were treated with high concentrations of glucose (30 mM) for 4 h, and then treated with BAIBA (10 μM) for 48 h in the presence of high glucose. To induce ER stress, HepG2 cells were incubated with thapsigargin (1 μM) or tunicamycin (3 μM) for 4 h in DMEM containing 10% FBS, and subsequently treated with different concentrations of (1, 10 or 100 μM) for 48 h in the presence of thapsigargin or tunicamycin^51^,^52^.

### Transfection with siRNA

HepG2 cells were plated on 60 mm plates on the day before transfection and grown to 30–50% confluence. Small interference RNA (siRNA) sequences against AMPK (20 nM) or a scrambled siRNA (20 nM) were transiently transfected into the cells using Lipofectamine (Invitrogen, Carlsbad, CA, USA) following the manufacturer’s protocols^53^. The transfected cells were washed with PBS (pH 7.4) 6 h after the transfection. The cells were treated with glucosamine (18 mM) for 18 h and followed by the treatment with BAIBA for 48 h in the presence of glucosamine. The sequences of AMPK-siRNA were listed as follows. Sense: CGGGAUCCAUCAGCAACUATT; antisense: UAGUUGCUGAUGGAUCCCGAT^54^.

### Flow cytometry analysis

Apoptosis assay was according to the manufactory’s protocols of FITC Annexin V kit (BD Biosciences, Franklin Lakes, NJ, USA). Briefly, cells were trypsinized and washed with cold PBS and incubated with FITC-conjugated Annexin V and propidium iodide. Cells were analyzed by flow cytometry^55^.

### ITT and GTT

To determine insulin tolerance and glucose tolerance, mice were fasted for 6 hours and overnight, respectively, and followed by intraperitoneal injection of insulin (0.75 units/kg body weight) or glucose (2.0 g/kg body weight), respectively. Blood samples were collected from tail veins, and blood glucose levels were measured using the blood glucometer (One Touch, Johnson & Johnson, USA) at 0, 15, 30, 60 and 120 min after the insulin or glucose injection^56^,^57^.

### Blood chemistry and Liver triglyceride levels

Serum insulin levels were determined with a rat/mouse insulin Elisa kit (EZRMI-13K; Millipore, USA)^58^. Fasting blood glucose levels, serum TG, TCH, FFA, LDL-C and HDL-C levels, as well as serum ALT and AST activity were measured with commercial kits (Jiancheng Bioengineering Institute, Nanjing, China). Hepatic TG content was determined with commercial kits (Applygen Technologies Inc., Beijing, China).

### Western blotting analyses

Protein extracts were electrophoresed, blotted, and then incubated with corresponding specific antibodies against PEPCK, G6pase, Akt, phospho-Akt (Ser473), IRS-1, phospho-IRS-1 (Ser307), phospho-IRS-1 (Tyr632), eIF2α, phospho-eIF2α (Ser51), JNK, phospho-JNK (Thr183/Tyr185), GRP78, ATF4, Bax, Bcl-2, CHOP, caspase-3, caspase-9, β-actin and GAPDH with appropriate secondary HRP-conjugated antibodies, and then developed. Antibodies against JNK and GAPDH bought from Abcam (Cambridge, England), and other antibodies were bought from Cell Signaling (Beverly, MA, USA).

### Real-time RT-PCR

Total RNAs from livers were isolated using Trizol reagent (Invitrogen, CA, USA). Real-time PCR was performed using a StepOnePlus Real-Time PCR System (Applied Biosystems, Foster City, CA, USA). All genes expression levels were normalized by GAPDH levels. The sequences of primers were listed in the online supplemental data (Supplementary Table S1).

### Histology and immunohistochemistry

Livers were fixed in 4% neutral buffered formalin. The tissues were embedded in paraffin, and sliced into 5 μm-sections. Hematoxilin and eosin (H&E) staining and Oil red O staining were used to detect lipid content in livers. Immunohistochemistry was used to evaluate CHOP expression in livers. The sections were incubated with anti-CHOP antibody (1:50, Cell Signaling Technology, Inc. Beverly, MA, USA).

### TUNEL assay

*TUNEL assay* was performed with the TUNEL Apoptosis Detection kit (Roche Applied Science, Mannheim, Germany). The numbers of TUNEL positive cells in liver sections were counted under light microscopy in 10 randomly selected fields (400× magnification).

### Chemicals

STZ, BAIBA, glucose, glucosamine, GalN, LPS, thapsigargin and tunicamycin were obtained from Sigma (St Louis, MO, USA).

### Statistical Analyses

Data are expressed as mean ± S.E. One-way and two-way ANOVA were used for data analysis of more than two groups followed by Bonferroni’s post hoc analysis. The criterion for statistical significance was set at P < 0.05 or P < 0.01.

## Additional Information

**How to cite this article**: Shi, C.-X. *et al*. β-aminoisobutyric acid attenuates hepatic endoplasmic reticulum stress and glucose/lipid metabolic disturbance in mice with type 2 diabetes. *Sci. Rep.* **6**, 21924; doi: 10.1038/srep21924 (2016).

## Supplementary Material

Supplementary Information
