# Supplementary material for: β-aminoisobutyric acid attenuates hepatic endoplasmic reticulum stress and glucose/lipid metabolic disturbance in mice with type 2 diabetes
Source: Sci Rep. 2016 Feb 24;6:21924. doi: 10.1038/srep21924 (PMC4764829; doi:10.1038/srep21924)
Supplement: Supplementary Information [file srep21924-s1.doc]

**β-aminoisobutyric acid attenuates hepatic** **endoplasmic reticulum stress and glucose/lipid metabolic disturbance in mice with type 2 diabetes**

Chang-Xiang Shi^1^, Ming-Xia Zhao^1^, Xiao-Dong Shu^1^, Xiao-Qing Xiong^1^, Jue-Jin Wang^1^, Xing-Ya Gao^1^, Qi Chen^2^, Yue-Hua Li^2^, Yu-Ming Kang^3^, Guo-Qing Zhu^1,2^*****

^1^Key Laboratory of Cardiovascular Disease and Molecular Intervention, Department of Physiology, Nanjing Medical University, Nanjing, Jiangsu 210029, China; ^2^Department of Pathophysiology, Nanjing Medical University, Nanjing, Jiangsu 210029, China; ^3^Department of Physiology and Pathophysiology, Cardiovascular Research Center, Xi'an Jiaotong University School of Medicine, Xi'an 710061, China

**
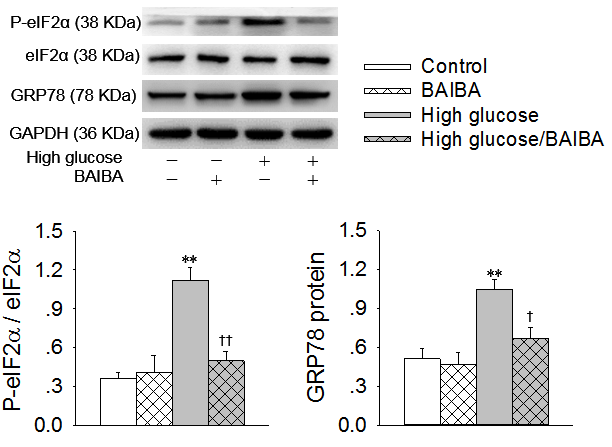
**

**Supplementary Figure 1** BAIBA attenuates High glucose-induced ER stress in HepG2 cells. The HepG2 cells were treated with high concentration of glucose (30 mM) for 4 h, and then treated with BAIBA (10 μM) for 48 h. The eIF2α phosphorylation and the GRP78 expression were used as the markers of ER stress. **P<0.01 vs. Control; †P<0.05 and †† P<0.01 vs. High glucose. n=4 for each group.

**
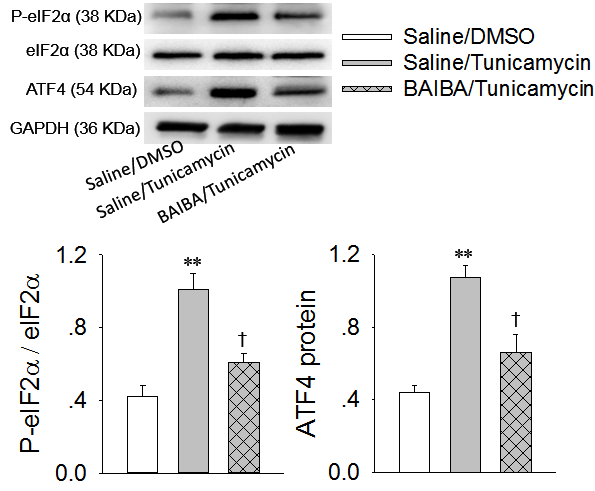
**

**Supplementary Figure 2** BAIBA attenuates tunicamycin-induced ER stress in the livers of mice. Mice were injected with saline or BAIBA (300 mg/Kg, i.p.) 0.5 h before DMSO or tunicamycin (2.5 mg/Kg, i.p.). The livers were collected 2 h after administration of DMSO or tunicamycin. The eIF2α phosphorylation and ATF4 expression were used as the markers of ER stress. **P<0.01 vs. DMSO; †P<0.05 vs. tunicamycin. n=4 for each group.


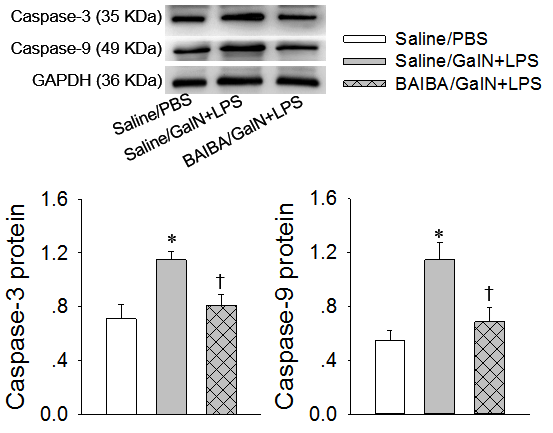


**Supplementary Figure 3** BAIBA attenuates GalN/LPS-induced apoptosis in the livers of mice. Mice were injected with saline or BAIBA (300 mg/Kg, i.p.) 0.5 h before PBS or GalN (700 mg/kg, i.p.) plus LPS (100 μg/kg, i.p.). The livers were collected 6 h after administration of PBS or GalN/LPS. Caspase-3 and caspase-9 expressions were used as the markers of apoptosis. *P<0.05 vs. PBS; †P<0.05 vs. GalN+LPS. n=4 for each group.

**
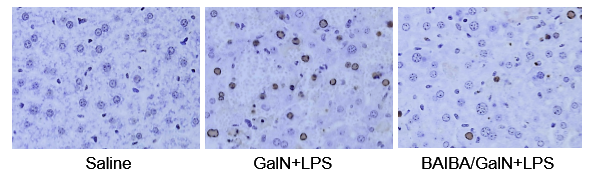
**

**Supplementary Figure 4** Images of TUNEL staining showing that BAIBA attenuates D-galactosamine/lipopolysaccharide (GalN/LPS)-induced apoptosis in the livers of mice. Mice were injected with saline or BAIBA (300 mg/Kg, i.p.) 0.5 h before PBS or GalN (700 mg/kg, i.p.) plus LPS (100 μg/kg, i.p.). The livers were collected 6 h after administration of PBS or GalN+LPS.

**
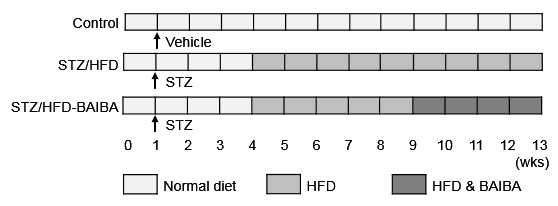
**

**Supplementary Figure 5** Schematic diagram showing the timeline of the experiment in three groups of mice.

**Supplementary Table S1 Primers for r**eal-time quantitative PCR analysis

|  | Primer | Sequence |
| --- | --- | --- |
| Srebp-1c | Forward | TGGAGACATCGCAAACAAG |
|  | Reverse | ACCAGTGGATGCAGGGAT |
| Fas | Forward | TTCCGTCACTTCCAGTTAGAG |
|  | Reverse | TTCAGTGAGGCGTAGTAGACA |
| Acc1 | Forward | TACCTTACAGTTCAGTCTCGGTG |
|  | Reverse | CTGCTTTCTTGGACAAATGG |
| Scd1 | Forward | CACCTGCCTCTTCGGGATTT |
|  | Reverse | TCTGAGAACTTGTGGTGGGC |
| Caspase-3 | Forward | CCTGGAGAAATTCAAAGGACGGG |
| Caspase-9 | Reverse  Forward  Reverse | GCATGGACACAATACACGGGATCT  CAGACCATCTACACATGCAG  CTCAAACAAAACCAACCAACC |
| GAPDH | Forward | TCAACGGCACAGTCAAGG |
|  | Reverse | ACCAGTGGATGCAGGGAT |
